# On-Site Detection of Volatile Organic Compounds (VOCs)

**DOI:** 10.3390/molecules28041598

**Published:** 2023-02-07

**Authors:** Ruben Epping, Matthias Koch

**Affiliations:** Division of Organic Trace and Food Analysis, Bundesanstalt für Materialforschung und -Prüfung, 12489 Berlin, Germany

**Keywords:** volatile organic compounds (VOC), on-site detection, mobile analytics, sensors, gas chromatography, mass spectrometry

## Abstract

Volatile organic compounds (VOCs) are of interest in many different fields. Among them are food and fragrance analysis, environmental and atmospheric research, industrial applications, security or medical and life science. In the past, the characterization of these compounds was mostly performed via sample collection and off-site analysis with gas chromatography coupled to mass spectrometry (GC-MS) as the gold standard. While powerful, this method also has several drawbacks such as being slow, expensive, and demanding on the user. For decades, intense research has been dedicated to find methods for fast VOC analysis on-site with time and spatial resolution. We present the working principles of the most important, utilized, and researched technologies for this purpose and highlight important publications from the last five years. In this overview, non-selective gas sensors, electronic noses, spectroscopic methods, miniaturized gas chromatography, ion mobility spectrometry and direct injection mass spectrometry are covered. The advantages and limitations of the different methods are compared. Finally, we give our outlook into the future progression of this field of research.

## 1. Introduction

Volatile organic compounds (VOCs) are chemicals with a high vapor pressure at room temperature and standard atmospheric pressure. The definition varies slightly among different industries, fields of research, countries and regulating bodies. It may include cutoff values for the vapor pressure (e.g., >0.01 kPa at 20 °C in the EU) [1] exclude non/low-reactive compounds (EPA, USA) [2], include a low water solubility or differs for indoor and outdoor air. Since the vapor pressure correlates with the boiling point, they may also be defined as compounds with an initial boiling point of less than 250 °C [1]. They are organic compounds including hydrocarbons, alcohols, aldehydes, organic acids. VOCs can be divided between biological and anthropogenic sources. VOCs can be further classified by their volatility, as seen in Table 1 [1].

Biogenic volatile organic compounds (BVOCs) can be emitted by plants, animals or microorganisms. They are the result of (secondary) metabolic or decomposition processes. Most prevalent BVOCs are terpenoids, especially isoprene, alcohols and carbonyls. Reasons for the release of BVOCs vary but are oftentimes linked to communication [3]. Examples are plants attracting insects to their flowers, plants defending against herbivores or animals deterring rivals from a territory. BVOCs make up the largest part of VOCs in the atmosphere. Here, they interact in different ways with atmospheric, mostly photochemical, processes. Most well-known is their interaction with atmospheric radicals [4]. Their lifetimes in the atmosphere can vary from hours to months.

Anthropogenic VOCs stem from fossil fuels, especially from incomplete combustions in vehicles, solvents used for paints, adhesives, cosmetics, coatings, plastics, furniture, aerosol products, perfumes, pesticides, flame retardants, cleaning supplies, industrial processes, tobacco smoke, cooking and a myriad of other sources [5].

Many VOCs can have a negative impact on the environment and constitute a considerable health risk [6]. Though the severeness of health risks varies greatly. In general, most of them do not pose a severe health risk but can cause problems in a long-term exposure scenario. As such VOCs can be toxic, carcinogenic, mutagenic, genotoxic and teratogenic to humans [7]. The concentration of these air pollutants can be higher in indoor than outdoor air, which is why indoor air is much more in the focus in this field of research and in regulatory efforts [8]. Environmentally VOCs can cause photochemical smog by reaction with nitrogen oxides (NO_x_) to form ozone [9]. Additionally, secondary particulate matter can be formed [10]. Implications of these can be reduced crop yields, interference with cloud formations and precipitation levels, damage to crops and buildings or increased global warming [11].

Therefore, the analysis of VOCs plays a crucial role in working towards sustainability and a better quality of life. Analysis of VOCs can help monitor and track air quality, identify sources of pollution and support efforts to reduce environmental exposure. Efforts to reduce human exposure to toxic VOCs can be supported by the assessment of indoor and outdoor air quality. Characterization of VOCs can help identify and reduce the use of harmful chemicals in consumer products, promoting more sustainable and environmentally friendly products. Analysis of VOCs in food products can help to ensure that they are safe for consumption and meet regulatory requirements.

Regarding analysis, the sampling of VOCs poses a challenge. Since samples are diluted by air, preconcentration is often needed [12]. This can be conducted by collection on sorption tubes or in cryo-traps [13]. Problematic with this could be the reactivity of VOCs either with surfaces or with each other as well as the accumulation of water. Samples could be compromised during collection, transport, or storage. Solid phase microextraction (SPME) may be used to collect trace amounts of VOCs [14]. To mitigate problems, the collection of samples can also be performed in gas sample bags, syringes or glass or steel containers without preconcentrating them. Sometimes it is necessary to store the samples at freezing temperatures to preserve them. Other times a certain amount of sample deterioration is unavoidable [15,16,17,18,19].

Other challenges in VOC characterization are found in the identification and quantification of them. Depending on the method of analysis the signal output may not be able to say anything about what specific VOCs are being measured. As a vast amount of different chemical compounds can be classified as VOCs the speciation of them can be challenging. Additionally, the response factors of a device towards these different compounds may vary greatly which makes quantification difficult as well. Another challenge for the quantification is the required low limit of detection. Many VOCs have very low concentrations in environmental samples and require highly sensitive analytical methods to detect. Additionally, interferences from the matrix can further complicate the quantification.

The most common measuring method for VOCs is gas chromatographic (GC) separation followed by different kinds/types of detection. Samples can be brought into the system directly, by thermal desorption or by solvent desorption. Numerous different applications are now known for this purpose [13,20,21,22]. The separation of components is performed on mostly nonpolar columns followed by a detector, e.g., a flame ionization detector (FID), electron capture detector (ECD) or other more specialized detectors [13,23]. Most methods, however, use mass spectrometers as detectors (GC-MS). Due to the volatile and reactive nature of some VOCs, it is often difficult to obtain stable reference standards to ensure the calibration of measurements [24,25]. These reference standards can be generated from gas tanks or via dynamic methods [24,26].

In summary, the established method of GC(-MS), while powerful and versatile, has some challenges and limitations. First is the preservation of the true sample at the sample collection state. Certain compounds may be too fragile to be collected, altered while stored, or not be collected selectively or without losses. In addition, both the sample and analysis stage take a lot of time and need to be performed off-line in the lab which further complicates the measurement and adds to their costs. This can be problematic whenever the results must be determined quickly and/or cost efficiently.

For those reasons, this review aims to present analytical methods beyond an off-line sample collection with a lab analysis by GC(-MS). Especially on-site, in situ and mobile analysis techniques are emphasized. We briefly describe the basic functionality of the methods and highlight notable publications and improvements in the last five years. We also focus on the analysis of VOCs in air rather than in liquid or solid matrices. While the topic of VOCs characterization could be sectioned in different ways, for example by means of the emitting source, the different chemical classes, fields of research that are interested in VOC or a classification based on harmfulness, etc., we chose to divide this review by the means of analysis. By this, we hope to give researchers inspiration for their approach to future works regarding the analysis of VOCs. This review paper is meant for newcomers to the field of VOC analysis that search for ways to tackle specific or general analytical challenges.

## 2. Fast/Screening Methods for on-Site/Mobile Analysis of VOCs

### 2.1. Nonselective Gas Sensors

There are several types of gas sensors that can be used to measure volatile analytes. These sensors are mostly used to measure the total VOC content due to their nonselective nature. Most of these sensors can usually just detect the presence and concentration of volatiles in the air but not speciate them. The most used types of sensors that can be included in this category are photoionization detectors (PID) electrochemical sensors (ECS) or metal oxide sensors (MOS).

#### 2.1.1. Photoionization Detectors

PIDs use light, typically in the UV range, to ionize gas molecules. The energy of the photons is enough to ionize most organic compounds but not the main constituents of air. Measurable compounds include almost all VOCs and some inorganic molecules such as ammonia and hydrogen sulfide but notably not methane and other very low molecular weight VOCs. The electric current produced by the ions on an electrode constitutes the detector output. They are commonly used to monitor the exposure of workers in different settings to VOCs.

Even though PIDs have been in use for decades, work on this technique continues to this day. Covington and Agbroke described a PID sensor that can discriminate between some VOCs and provide at least some compositional information [27]. Pang et al. explored the use of PIDs to replace an FID as a GC detector. They found it to perform similar with the advantage of better portability and independence from hydrogen. Additionally, options for the use of different lamps were explored [28]. The possibility of a standalone PID to discriminate between volatiles was investigated by Spadi et al. They were able to classify different rosemary species by looking into the temporal data acquisition of the PID. The temporal kinetics of the VOC emission were found to be distinct for each species and a ‘fingerprint’ for each variety could be obtained [29].

Similar to PID is a flame ionization detector (FID), although an FID uses a hydrogen flame to ionize the VOCs. FIDs are most commonly known as GC detectors but can also be utilized as stand-alone instruments. In contrast to a PID, an FID responds better to carbon chains while a PID is better suited for the detection of functional groups [30]. The signal is proportional to the number of non-oxidized carbon atoms. Both detectors are commonly used as total VOC detectors. However, even though FIDs are still frequently used in total VOC analysis innovation has shifted away from this well established and understood technique in recent years. One major drawback for their usability is the requirement of hydrogen gas.

#### 2.1.2. Electrochemical (Amperometric) Sensors

Electrochemical sensors (ECSs) measure the current of a redox reaction in which a charge is transferred from an electrode to the analyte in an electrochemical cell of a varying constitution. In most ECSs a measuring cell is compared to a reference cell. In the measuring cell the VOC analytes diffuse into the cell (and the electrolyte) through a membrane. While in principle also nonselective, an ECS can be optimized for specific analytes. This can be performed by altering the membrane material, the electrode materials, the electrolyte, or the electrical state of the cell.

Silverster described the benefits of using ionic liquids as electrolytes in ECSs. They have a wide potential range and the capability to improve the lifespan of sensors in dry conditions. They also facilitate the miniaturization of the devices [31].

Miha et al. highlighted the use of polypyrroles as a sensor material in electrochemical sensors. They describe the effects of doping, surface modification, side-chain selection and other variants and elaborate on their utilities [32].

Kumar et al. report on the progress and challenges of using metal organic frameworks in electrochemical VOC detectors [33]. They discuss strategies of doping, tagging/functionalization, and post-synthesis modification to maximize performance.

#### 2.1.3. Metal Oxide (Potentiometric) Sensors

Metal oxide sensors (MOS) sensors are composed of a metal oxide thin film. They are the most common and widely used VOC sensors. The resistance and conductivity are altered by ambient gases that desorb onto its surface [34]. Depending on the metal oxide, oxidizing, or reducing compounds can be measured. The working principle is not fully understood but depends on the interaction of the VOC compounds with chemisorbed oxygen which either liberates or removes electrons from the semiconductor surface. N-type MOS (e.g., TiO_2_) experiences a decrease in conductivity from oxidizing and an increase from reducing VOCs. P-type sensors (e.g., NiO) work consequently the opposite way [35]. The performance of MOSs can be influenced by, among others, their composition, surface area, doping level, temperature, humidity, or morphology [36].

Consequently, a lot of research in the past years focused on the fabrication methods and nanostructured MOSs. Some nanostructured MOSs showed significantly enhanced selectivity and were able to operate with lower temperatures [35,37,38,39,40,41,42]. Acharyya et al. were able to discriminate different VOCs with a tin-oxide-based hollow sphere MOS by calculating the kinetic properties of the analytes interaction with the active surface [43]. A significant improvement of selectivity and sensitivity was achieved by Fois et al. by using metal oxides doped with rare earth elements [44]. An overall increase in performance of MOSs was reported by Pargoletti and Cappelletti by coupling them with innovative carbonaceous materials [45]. Baur et al. were able to show hidden potential in MOSs by using them in a temperature cycled operation and data-based models trained with advanced machine learning [46]. Gao et al. were able to improve the fabrication of MOSs by synthesizing hierarchically porous metal oxide nanostructures [47].

All three types of these nonselective sensors mentioned to this point have the advantage of being typically relatively cheap, small (mm to cm range) and commercially available. Their limit of detection (LOD) ranges from mid ppb to ppm levels. PIDs are the simplest devices with a relatively low LOD, high measuring range and short measuring time [48]. However, not all VOCs are detectable. Halogenated species for example cannot be ionized. In comparison, ECSs are less sensitive and have a more limited detection range but respond to a broader range of analytes. A measurement typically takes >2 min due to the slow diffusion process. They may also need some humidity to work. On the other hand, they can be better tuned to measure specific VOCs and they require very little energy to run [49]. MOS sensors are typically very small and light. Their LOD differs vastly for different compounds. Their selectivity may be influenced by dopants or filters. Since they usually function only in higher temperature, they have a higher power consumption. The adsorption and desorption of VOCs onto the surface is additionally very slow which leads to measurement times of up to 1 h. They also show a response to some inorganic gases such as CO or NOx. The sensor is additionally affected by temperature and humidity. In general, MOS sensors are not stable for a very long time [49].

#### 2.1.4. Pellistors, Surface Acoustic Wave, Quartz Crystal Microbalance and Other Sensors

One more sensor that belongs to the group of nonselective VOC sensors are pellistors or thermal sensors. These measure the changing resistance of a catalytically coated ceramic while volatiles that are combustible move towards them by diffusion. The heat increase through the combustion reaction leads to an increase in electrical resistance. Due to this nature, they are used to detect explosives though they have a high LOD (high ppm range) [50]. Pellistors are among the oldest sensor technologies still in use.

Besides these more established and widely used sensors, there is a wide range of emerging sensors that may play an increasingly important role in the future. These techniques include, for example, surface acoustic wave (SAW) sensors. Within the SAW, an acoustic wave travels through an adsorbent polymer film on a piezoelectric substate [51]. When a VOC is adsorbed onto the film the mass of the film increases resulting in a small change in phase of the wave relative to a reference. For this, films with different affinities can be used [52].

Gao et al. proposed a variation of a SAW named dual transduction SAW. By detection variations in both the mass and resistance in the sensing material and exploiting the relationship between them, they were able to identify different VOCs [53]. The sensitivity of a SAW sensor could be considerably increased by Viespe et al. by embedding nanoparticles in the polymer sensing film [54]. Similar effects were seen by Kus et al. by using functionalized gold nanorods [55]. A review for sensitive materials and coating technologies was performed by Palla-Papavlu et al. [56].

Another sensor that works on a similar principle is a quartz crystal microbalance (QCM). A voltage is aplite to a quartz crystal causing it to oscillate (reverse piezoelectric effect). The crystal is coated with a film like that of a SAW. When a VOC adsorbs onto the surface, the change in mass results in a change in frequency which is recorded [57,58,59].

Due to their viscoelastic nature ionic liquids have gained much interest as a coating for QCMs in recent years [60,61].

Besides these types, there are several other detector types being researched now that can be counted towards the class of microelectromechanical systems (MEMS), subcategories of them or sensors already mentioned here. The basic mechanism for all these sensors is an active layer that interacts with the VOCs and a transduction mechanism that translates that interaction into a signal. The transduction mechanism can be optical, acoustic, calorimetric, amperometry, conductometric, potentiometric or biological [48]. Since it would be impractical to cover all of them in this work, we will leave it in the above description of the most common and/or promising types.

### 2.2. Electronic Noses

An electronic nose (EN) or e-nose refers to the principle of a sensor array. Several different simple and low-cost detectors (see Section 2.1) are bundled together to form the array [62]. While an individual sensor is rather nonselective, each different sensor has a slightly different selectivity towards a certain VOC compound. Through pattern recognition algorithms the information from all sensors is combined to form a fingerprint of an odor. These devices are usually used to compare odor samples (comprised of many VOCs) with a reference or with each other [63]. This may be utilized in food safety to identify off-odors, classify different foods or food from different sources [64]. Other fields of use are agriculture, forestry, military and security, medical or industrial applications. Although low-cost sensors are used to build electronic noses, the cost and size of them are considerably higher than that of just the gas sensors due to the advanced computational and software requirements. E-nose systems are among the most promising technologies for odor discrimination because they mimic our own olfactory system. The sensor array mimics our nose while the data analysis takes the equivalent role to our brain. The data analysis is performed by utilizing different machine learning algorithms such as pattern recognition, principle component analysis, linear discrimination analysis and others with the goal to form artificial neural networks [64].

ENs are most prominently used for the authentication and quality control of food [65,66,67,68,69,70,71]. Since the use of an EN requires no sample preparation and the calibration can be performed using just a reference sample this technique seems well suited for this task.

The detection of diseases in particular by analyzing exhaled breath is becoming an increasingly researched topic [72,73,74,75,76,77]. As it is often not feasible and impractical to utilize techniques such as GC-MS for breath analysis, focus on this field has shifted towards ENs. Since human breath can contain up to 3000 VOCs [72], formed mostly by metabolic processes, it is believed that an analysis can led to information on a person’s health. The recent applications for ENs in both food and disease detection are too numerous to mention individually but can be looked up in the cited literature above.

One aspect that may have prevented the widespread adoption of ENs is portability. The device needs to be small, light, low power consuming and fast in response to be applicable in the field and at the points of interest [78]. Wojnowski et al. constructed a particularly portable and modular ENs for the purpose of food analysis [65]. Hou et al. developed a handheld EN to identify liquors [79]. Huang et al. designed and validated a portable, battery-powered EN based on 10 MOSs and machine learning algorithms for the detection and classification of VOCs [80]. Matatagui et al. were able to construct a portable, low-cost, battery powered EN based on SAW sensors for the detection of BTX in air [81]. The main challenge in these advancements lays in the manufacturing of the appropriate electronic parts (acquisition and transmission module), the signal processing and the software to process the gathered data. The detection, classification, and prediction can be improved by using artificial intelligence in an EN [82].

### 2.3. Spectroscopic Methods

Optical sensors in general are more selective than simple gas sensors. While not molecule specific, they still offer better selectivity, a lower instrumental drift and faster response with the additional benefit of being non-destructive. On the other hand, they are also larger and costlier. Since the measurement is based on the direct measurement of physical properties of molecules it is reliable and self-referenced [83].

#### 2.3.1. Nondispersive Infrared Sensors

Nondispersive Infrared Sensors (NDIR) measure the absorption of infrared light of components in the gas phase. The IR spectral region of 700 nm–14 µm causes molecular vibrations and rotations in analytes. In contrast to traditional IR spectrometers no diffraction grating, or prism is used to select a spectrum range [84]. Instead, an optical filter is used to select regions from a broadband source for analysis. With this semi-selective method, the decrease at wavenumber regions, which represent specific oscillation frequencies, can be observed. These typically represent chemical functionalities/bonds of the analytes. All types of compounds, including inorganic gases can be detected with these techniques. While some selectivity is given, the absorption of a specific compound may be overlaid by the absorption of another compound with the same molecular bonds [85]. While this technique is applicable for VOCs, it is most used to detect small gas molecules such as H_2_S, CH_4_, CO_2_, CO, NO, CH_2_O, NO_2_ or SO_2_.

TAN et al. reported on multiplexed NDIR gas sensing platform utilizing a narrowband infrared detector array. They achieved multi-gas sensing with nanoantenna integrated narrowband pyroelectric detectors instead of multiple pairs of bulky and expensive bandpass filters and detectors [86]. A similar system was shown by Xu et al. to be able to analyze multiple automobile exhaust gases simultaneously [87]. Esfahani et al. demonstrated that NDIRs can also be used as sensors in an electronic nose, overcoming common issues such as sensor drift, poor repeatability, lack of robustness, insufficient replicability and temperature and humidity effects [88].

#### 2.3.2. UV Spectrometers

Besides NDIR, there are also several types of UV spectrometers that can analyze UV-active VOCs (e.g., aromatics). Absorption occurs when a valence electron is excited from a lower to a higher level by photons passing through the analyte. The energy of the photons absorbed matches the energy difference of the two electron levels and therefore is somewhat selective. The quantification is (in most cases) based on measuring the transmittance. Due to the simplicity of the principle this technique has been a staple in analytical chemical laboratories for decades. UV means the wavelength of the electromagnetic radiation spectrum between 180 nm and 400 nm. Miniaturization of the three main components can be achieved by utilizing LEDs as light sources, hollow core waveguides as a gas cell and photodiodes as detectors [83]. For gashouse analytes, UV spectroscopy is mostly used for the characterization of ozone, nitrogen oxides, sulfur dioxide and aromatics.

Hue et al. were able to build an absorption spectrometer capable of analyzing BTEX (benzene, toluene, ethylbenzene and xylenes) simultaneously to assess the quality of indoor air. They achieved this by combining five sensors containing nonporous disks with pore sizes tailored to entrap target analytes [89].

The emergence of deep UV emitting diodes could potentially expand the use of this technology over a wide range of fields [90].

#### 2.3.3. Chemiluminescence

Chemiluminescence (CL) describes a chemical reaction that emits light. While there are several liquid-based chemiluminescence reactions, the reaction with ozone is one gas phase reaction that can be used in VOC analysis [91]. Ozone can react in a chemiluminescence reaction with NO (used in car exhaust measurements), with reduced sulfur compounds (used in the study of the atmospheric sulfur cycle) and with compounds containing double bonds [92] (used in the study of isoprene emissions from plants [93]) [94]. Since this emission has a very low background and the wavelengths for each type of reaction differ, this type of detector can achieve very low limits of detections in the single digit ppt range. A CL detector can be used as a stand-alone device or as a sulfur or nitrogen specific detector in conjunction with a GC where all compounds are reduced in a hydrogen flame before the detector.

Ohira et al. used this principle to measure isoprene, which is involved in the biosynthetic pathway to cholesterol, in human breath [95]. Mukosera et al. were able to detect dinitrosyl iron complexes, important intermediates in the metabolism of NO with ozone chemiluminescence [96]. Zhao et al. introduced a chemiluminescence method to determine chemical oxygen demand in waters as an especially environmentally friendly and rapid method [97]. Similarly, Matsumoto was able to measure the total ozone reactivity stemming from BVOCs in forest air [98]. Conversely the chemiluminescence reaction of ozone can also be used to measure ozone concentrations for example in the atmosphere when using isoprene gas as a reaction partner [99,100].

### 2.4. Miniaturized GC

There has been a lot of interest in miniaturizing classic GC instruments for many years and various fields. This can be achieved to varying degrees, though the nomenclature is not consistent [101]. “Compact” GC instruments are smaller versions of lab instruments [102]. They performed about the same as their full-size equivalents, but consume less power, materials, and space. They are not as mobile though and are best suited for normal or temporary stationary laboratories. Instruments labeled as “portable”, or “field” are of even lower weights and suited for on-site analysis. Devices labeled as “µGC”, “handheld”, “pocket”, or “personal” are the smallest category that usually weights less than one kilogram [103]. These are chip-based GCs machined on silicon wafers. In general, the smaller the instrument the more limited the performance is [104].

All main components of a GC system, the injector, column, and detector, are shrunken to achieve better portability [22]. Due to low ambient concentrations, a simple preconcentration column with thermal desorption is most common as an injection unit [21,103]. Traditional columns may be replaced by etched channels on a semiconductor chip or a multiple capillary column. Possible detectors are miniature versions of classic GC detectors or may utilize one or more detectors mentioned in Section 2.1 [105]. To achieve true portability, these devices can be run on batteries and may use air as a carrier gas or use a gas cylinder. Additionally, smaller pumps and valves are utilized. While mini/µ-GCs are certainly more expensive and complex than simpler gas sensors the main advantage to utilize them is the much greater capability to be selective towards specific target analytes.

Rodríguez-Cuevas et al. were able to manufacture a novel gas preconcentrator with which they were able to analyze BTEX components at ppt level concentrations [106]. Similarly, Zamponi et al. combined micromachined GC components with an MOX detector and their own innovative preconcentration material. The preconcentration column was based on a silicon cartridge machined from a waver filled with quinoxaline-bridged cavitand. This stationary phase was able to interact with aromatic VOCs by weak CH–π interactions [107]. You et al. reported on a real time monitoring portable GC for VOCs. By using compressed air as mobile phase with a PID detector and a carbon nanotube sponge preconcentrator they were able to analyze samples at sub ppb level concentrations in <10 min [108]. Even two-dimensional GC in a portable format was achieved in recent years. Lee et al. used this fully automated technique to analyze VOCs released from paints in indoor air [109]. An example of what can be achieved in miniaturization was published by Wang et al. who build a belt-mounted GC. The system was intended for VOC monitoring in industrial workplace environments. The battery-powered device was able to characterize typical VOC mixtures in the ppb concentration level range in less than five minutes [110,111]. The capabilities of a µPID detector were greatly improved by Li et al. Results show similar performance to a benchtop GC-FID and were demonstrated on car exhausts and breath analyzers [112].

### 2.5. Portable Mass Spectrometry and Ion Mobility Spectrometry

Mass spectrometry (MS) is often seen as the gold standard in analytical chemistry providing information about molecular weights and chemical structures. High resolution MS can provide information down to the molecular formula of a compound. Traditional lab bound MS systems are heavy and consume a lot of energy. While there are a great number of different techniques out there, all are comprising the three main components of an ion source, mass analyzer and detector [113]. While there was a lot of innovation in terms of the ion source, what is holding portable systems back is mainly the mass analyzer due to the need for vacuum conditions and space to separate ions. For a real-world field application, it is also necessary to cut down on the complexity of operating such a system [114,115]. Despite these limitations, there are several examples where (somewhat) portable MS systems were used to detect VOCs. While there are portable versions of GC-MS devices that are intensively researched and that are already being commercialized, we wanted to focus on standalone MS/IMS methods for this section.

#### 2.5.1. Direct Injection Mass Spectrometry (DIMS)

A promising variant of utilizing MS for the analysis of VOCs are direct injection mass spectrometers (DIMS). These promise the ability to identify and quantify VOCs without a prior separation and in some cases even without proper calibration [116]. Hereby more specialized and soft ionization techniques are utilized in combination with a high-resolution mass analyzer (HRMS). The oldest technique to be used for this is atmospheric-pressure chemical ionization (APCI). Nowadays, new ionization methods have been developed to better control the ionization. Prominent instruments of this class are proton-transfer-reaction mass spectrometry (PTR-MS) [117] or selected ion-flow-tube mass spectrometry (SIFT-MS) [118]. Additionally, direct analysis in real time (DART) and soft ionization by chemical reaction in transfer (SICRIT) gained traction in recent years. While these instruments are not as mobile as handheld devices, they are at least mobile enough to be transported by a vehicle. As the name suggests, the DIMS instrument can analyze gas samples without any sample preparation [119]. The quantitative ion generation is achieved through controlling the ionization by first generation only precursor ions such as H_3_O^+^ which in turn ionize the volatile sample molecules. Some techniques can also use other precursor ions such as NO^+^ and O_2_^+^. Since this is a soft ionization which generates mostly intact molecular ions and the sample in most cases is also already diluted, the ionization can occur quantitatively [120]. One more advantage of these methods is the analysis time which can be in the seconds range while still ensuring great selectivity and sensitivity. In comparison to GC-MS which usually takes at least 30 min, the online analysis of DIMS techniques shortens the time of analysis considerably.

The usefulness of PTR-MS to monitor dynamic changes in food samples was highlighted by Majchrzak et al. [121]. The VOC profile can be used to indicate quality, shelf-life, or authenticity and track changes during production, preparation and storage. In the task of classification of food samples DIMS methods now outperform E-Nose systems which was highlighted by Deuscher et al. in the case study of dark chocolates organoleptics [122]. The usefulness of DIMS techniques in fast and sensitive flavor release analysis was reviewed by Taylor et al. [123]. They especially investigated the relationship between food aroma composition and flavor perception. Mazzucotelli et al. additionally highlight the compliance of PTR-MS (and others) with the guidelines of green analytical chemistry. They highlight recent applications in food consumption and sensory, bioprocess monitoring, traceability, quality control, and high-throughput food volatilome phenotyping [124].

Besides food analysis, DIMS can also be of great value in studying atmospheric VOCs [125] and VOCs in environmental matrices. Liu et al. utilized the fast measurements and mobility of PTR-MS to characterize indoor VOC emissions in a residential house. Doing so they were able to gain space- and time-resolved information on emission sources and concentrations [126]. The sensitivity of a PTR-MS system is showcased by Georgios et al., who identified VOC markers for volatile chemical products such as personal care products, fragrances adhesives or coatings in an urban environment [127]. They could measure the magnitude of different emission sources relative to motor traffic and draw correlations to population densities. Wu et al. investigated the contribution to oxygenated VOC compounds in the tropospheric atmosphere in an urban setting [128].

Another area of interest for DIMS is volatolomics concerning the study of VOCs released from humans, plants, insects or microorganisms [129]. Ghislain et al. described the ionization of carboxylic acids, which are involved in many biochemical processes, with SIFT-MS. The reaction pathways and kinetic constants and limits of detections were calculated. An intensely studied subject is the diagnosis of disease through breath analysis instead of other more invasive studies [130]. An example of the use of PTR-MS in this field is the investigation of cervical cancer markers by Zhou et al. [131]. While this route of diagnostic is not widely in use yet, many experts expect a breakthrough in this area to come with these new analytical tools soon. Since results of this type of investigations are often hard to replicate, Henderson et al. started the “peppermint initiative”. The initiative seeks to disseminate a standardized experiment that allows comparison of breath sampling and data analysis methods and also to share a set of benchmark values for the measurement of VOCs in breath [132].

#### 2.5.2. Ion Mobility Spectrometry (IMS)

Ion mobility spectrometry (IMS) can be seen as a hybrid between GC and MS. The big difference to MS is that the ion separation takes place under atmospheric pressure instead of in a vacuum. The Ions are dragged through a gas (usually air) and are separated according to their mobility. The mobility is a function of their mass to charge ratio and their shape [133,134,135]. When combined with a radioactive ^3^H electron emitter, an X-ray as the ionization unit or atmospheric pressure chemical ionization (APCI) the technique can be especially mobile and implemented in the form of handheld devices. IMS can be used as a stand-alone method or in conjunction with GC to help with the competing ionization process and add orthogonality [136].

A key component of an IMS is the drift tube. Ahrens et al. presented an easy-to-manufacture, miniaturized drift tube integrated into a stand-alone battery-powered mobile device [137]. Recently, Fulton et al. showed that a handheld IMS can be used to detect the drug fentanyl by measuring N-phenylpropanamide vapors without the need of physical contact with the highly potent substance [138]. Ratiu et al. used an aspiration IMS to sense metabolic VOCs from bacteria for discrimination purposes [139]. A statistical approach was used to process the IMS fingerprints. Guo et al. used both GC-MS and GC-IMS to investigate VOCs in oolong teas. They found 27 VOCs that were solely identified by IMS which highlighted the benefits of IMS also in combination with MS methods [140].

## 3. Discussion

The task of on-site detection of VOCs is of interest in many different fields of research. Among them are food and fragrance analysis, environmental and atmospheric research, industrial applications, security or medical and life science. All these applications and ever tightening regulatory demands for air quality induced a demand in the research of better analytical tools. With technical progress more and more innovations are emerging. While we gave a brief overview of the most relevant, meaning most used, best performing or intensely researched methods, there are many more emerging technologies out there. To help evaluate the progress and determine which technologies might gain future traction or are suitable for a defined purpose, criteria for what makes a good analytical technique can be found in Table 2 [141].

Nonselective gas sensors have made considerable progress in recent years in terms of sensitivity. While still relatively poor in selectivity, they also became cheaper. Due to their great portability, they are still best suited for many on-site applications. The drawbacks that remain are often a poor lifetime and instrumental drift. Depending on the method, data processing can also be challenging.

Spectroscopic methods are better suited in this area. However, they are not as portable and more expensive. While more selective than sensors, they are limited in terms of possible target analytes. The signal can at most times be easily translated into a readout.

Through the combination of multiple detectors E-Noses are able to produce fingerprints of complex VOC mixtures. This ability can be leveraged in many real-world applications due to their similar functioning to a human nose. However, they are not capable of identifying and qualifying single VOC compounds in a mixture.

For this purpose, IMS and DIMS technologies are well suited. This performance, however, comes with the drawback of limited portability and high cost.

The capabilities of the analytical methods mentioned in this article are summarized in Table 3. Here, suitable analytes as well as advantages and limitations of each method can be found.

**Figure 1:**
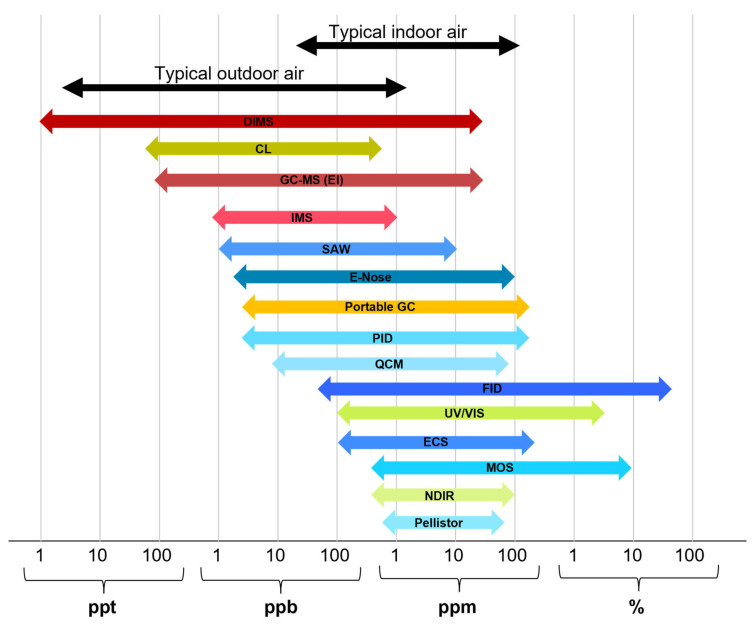
Measurement range of the analytical methods presented in this article compared to typical concentrations of VOCs in indoor and outdoor air. The given ranges are approximations that were compiled from all literature references cited in this article and represent the average properties of the methods which may not be true for every single device that belongs to them.

One of the most important criteria and often limiting factor for the use of VOC analytical techniques are the LODs and measurement ranges. In Figure 1, the dynamic ranges of all methods presented in this article are compared with each other and typical concentration ranges for VOCs in indoor and outdoor air. The given ranges are approximations that were compiled from all literature references cited in this article and represent the average properties of the methods which may not be true for every single device that belongs to them. These values can also be influenced by factors such as humidity temperature and others.

## 4. Future Directions

In the field of VOC analysis, we see a clear trend away from discontinuous measurements with a separate sample collection and/or enrichment and measurement off-site to a continuous measurement on-site. A further trend is the move from a separation followed by a detection step (see GC-MS) to a direct detection. This simplifies and speeds up the analysis which enables time and special resolved characterizations but comes with its own challenges. This can also be expressed by moving from a “sample to the lab” to a “lab to the sample” approach.

The main challenges with this are the low concentration of analytes, interferences from the matrix and other sample components, higher costs, and the instability of the used instruments. While an off-site GC-MS analysis is still the gold standard in this field, this method is also quite expensive, time consuming and requires specialized personnel. Depending on the requirements of analysis, we see three directions for the future of VOC analysis:

First, for simple use cases such as workplace exposure monitoring more and more semi-selective gas sensors will be utilized. With great improvements in selectivity and sensitivity due to novel coating materials for active layers and transduction mechanism as well as better operating stability and durability, sensor devices will soon be able to be used in more and more situations.

Second, for more advanced task such as the authentication of food- and aroma-sensing miniaturized GC devices with general purpose sensors as detectors as well as E-Nose devices will be used. The miniaturization and simplification of GC will go on from µGC to microfabricated microfluidic devices which are already subject to intensive research today. Preconcentration will not be necessary anymore and air will simply be used as the mobile phase. The capability of E-Nose devices depends largely on the sensors used in the array and the signal processing. Besides the above-mentioned improved sensors E-Noses can also use optical and mass selective detectors. These have the advantage of being more robust and the experience of less drift due to environmental changes. The applications these technologies will be used for do not require the precise knowledge of the concentration of individual VOCs. Instead, the use cases will mostly compare measurements with databases of fingerprints. All this will lead to a very simple operation of the devices where the end user will only receive a yes/no or good/bad output from the device.

Lastly, for the more advanced measurement requirements in research and industry DIMS, IMS or hybrid technologies (IMS separation followed by MS detection) will be used. This will lead to excellent selectivity and sensitivity, very fast response times and good quantification capability. In comparison to GC-MS, the time and expertise of the user needed will be decreased while not compromising the performance. The drawback of only limited portability, mostly due to the vacuum needed, as well as the high cost of the device will remain for the near future, but we can expect improvements also in this area.

## Figures and Tables

**Table 1:** Classification of VOCs according to boiling points.

Description	Boiling Point Range (°C)
Very volatile compounds (VVOC)	<0 to 50–100
Volatile organic compounds (VOC)	50–100 to 240–260
Semi-volatile organic compounds (SVOC)	240–260 to 380–400

**Table 2:** Criteria for analytical technique in the characterization of VOCs.

Sensitivity	Change of Signal Per Analyte Concentration
Selectivity	Response towards different analytes
Stability	Reproducibility of results over time
Operating conditions	Temperature range, humidity, and other conditions in which the system can operate
Response time and Frequency	Time from start of measurement to signal generation and number of measurements in a given time
Limit of detection (LOD)	Lowest concentration of analyte that can be detected
Dynamic range	Concentration range in which the method can be applied
Resource requirements	Consumption of electricity, gases, materials or other resources
Portability	Ranging from wearable to stationary
Cost and maintenance	Price for the device and operation
Accuracy, precision and robustness	Often dependent on calibration requirements, instrument drift and changing operating conditions
Price	Cost for the device and/or measurement
Data extraction	Ease of getting a readout from the measurement

**Table 3:** Methods described in the article with corresponding suitable analytes and advantages (+) and limitations (-) of them. This list was compiled from all literature references cited in this article and represent the average properties of the methods which may not be true for every single device that belongs to them.

Method	Selectivity/Analytes	Major Advantages/Limitations
PID	VOCs with the right ionization potential	+ instantaneous response + constant operation + inexpensive + simple to use + high measuring range + high sensitivity to aromatics - nonselective - halogenated species not detectable
ECS	Most VOCs	+ broadband sensors + low power consumption + durable (not poisoned by other gases) + low costs - limited sensitivity and detection range - limited temperature range - limited shelf life - large
MOS	Oxidizing compounds and most reducing compounds	+ good durability + small + low cost + easy to use + commercial production - cross sensitivity of VOCs and inorganic VCs - influenced by humidity - high working temperature - sulfur poisoning - not very flexible
Pellistors	Combustible volatiles	+ small + inexpensive - low selectivity - high LOD - sensitive to environment changes - baseline drift
SAW	Depended on the film material on the sensor	+ response to nearly all gases + small + sensitivity +Low power consumption + high sensitivity + fast response + long lifetime - still limited selectivity - temperature sensitive - signal-to-noise ratio - noisy - poor reproducibility
QCM	Depended on the film material on the sensor	+ sensitivity + fast response + good sensitivity + good reproducibility - limited selectivity - signal-to-noise ratio - humidity/temperature sensitive - limited measurement range - complexity
E-Nose	Almost all volatile compounds, depending on the sensors used in the array	+ fingerprinting specific odors comprised of multiple compounds + good selectivity - more expensive than the sum of individual sensors - quantification difficult
NDIR	IR absorbing VOCs (+ small inorganic compounds)	+ fast response + sensitivity + selectivity + stability + portable + insensitive to environmental changes + low power consumption + non-destructive - difficult to miniaturize - expensive - sensitivity - interferences - higher energy consumption than sensors
UV	All UV absorbing VOCs	+ sensitivity + selectivity + stability + very fast response + insensitive to environmental changes + non-destructive - difficult to miniaturize - expensive
CL	NO, reduced S-compounds, double bond compounds	+ sensitivity + selectivity + very fast response + low sensitivity to environmental changes + stability - need for ozone gas
µGC	Basically, all volatile analytes, depending on detector	+ good selectivity + high sensitivity + variability (e.g., stationary phase) + fingerprinting + good portability - discontinuous measurement - instrument maintenance
DIMS	All VOCs with proton affinity > precursor ion (mostly H_3_O^+^)	+ good selectivity + very good sensitivity + very fast response + simplicity (compared to other MS techniques) - limited portability - very expensive - very high energy consumption (plug bound)
IMS	All VOCs depending in ionization source	+ selectivity + sensitivity + portable + low power consumption + fast response - expensive - high energy consumption (battery possible)

## Data Availability

Not applicable.
